# Supplementary material for: Aspirin or enoxaparin for VTE prophylaxis after primary partial, total or revision hip or knee arthroplasty: A secondary analysis from the CRISTAL cluster randomized trial
Source: PLoS One. 2024 Apr 16;19(4):e0298152. doi: 10.1371/journal.pone.0298152 (PMC11020928; doi:10.1371/journal.pone.0298152)
Supplement: Attachment — Submitted filename: Authors Response Document.docx [file pone.0298152.s003.docx]

2^nd^ January, 2024

Dear Professor Fukumoto,

**RE: PONE-D-23-32485, “Aspirin or Enoxaparin for VTE Prophylaxis after Primary Partial, Total or Revision Hip or Knee Arthroplasty: A Secondary Analysis from the CRISTAL Cluster Randomized Trial”**

We thank you for your suggested revisions to the above manuscript. The authors’ responses to each question or comment from the reviewers (which are italicised) are listed underneath each comment. Relevant excerpts from the manuscript have been included and indented. New additions to the manuscript are underlined and those that have been deleted have been shown in strikethrough font. The section and page number of each change has been referenced.

Along with this response document, we have also included a “Tracked Changes” manuscript and a “Clean” manuscript in our revision submission.

**JOURNAL REQUIREMENTS:**

1. *Please ensure that your manuscript meets PLOS ONE's style requirements, including those for file naming. The PLOS ONE style templates can be found at*

[*https://journals.plos.org/plosone/s/file?id=wjVg/PLOSOne_formatting_sample_main_body.pdf*](https://journals.plos.org/plosone/s/file?id=wjVg/PLOSOne_formatting_sample_main_body.pdf) *and*

[*https://journals.plos.org/plosone/s/file?id=ba62/PLOSOne_formatting_sample_title_authors_affiliations.pdf*](https://journals.plos.org/plosone/s/file?id=ba62/PLOSOne_formatting_sample_title_authors_affiliations.pdf)

We have ensured that the manuscript meets PLOS ONE’s style requirements.

2. *Thank you for stating the following financial disclosure:*

*"The trial was funded by an MRFF Grant provided by the Australian Federal Government (grant number 1152285)"*

*Please state what role the funders took in the study.  If the funders had no role, please state: "The funders had no role in study design, data collection and analysis, decision to publish, or preparation of the manuscript."*

*If this statement is not correct you must amend it as needed. Please include this amended Role of Funder statement in your cover letter; we will change the online submission form on your behalf.*

We can confirm that the funders had no role in the study design, data collection and analysis, decision to publish or preparation of the manuscript.

We have included this in the “Role of Funder” statement in our cover letter.

3. *Thank you for stating the following in your Competing Interests section:*

*"NO authors have competing interests"*

*Please complete your Competing Interests on the online submission form to state any Competing Interests. If you have no competing interests, please state "The authors have declared that no competing interests exist.", as detailed online in our guide for authors at* [*http://journals.plos.org/plosone/s/submit-now*](http://journals.plos.org/plosone/s/submit-now)

*This information should be included in your cover letter; we will change the online submission form on your behalf.*

We can confirm that the authors have no competing interests to disclose. We have now included this statement in our cover letter.

4. *In your Data Availability statement, you have not specified where the minimal data set underlying the results described in your manuscript can be found. PLOS defines a study's minimal data set as the underlying data used to reach the conclusions drawn in the manuscript and any additional data required to replicate the reported study findings in their entirety. All PLOS journals require that the minimal data set be made fully available. For more information about our data policy, please see* [*http://journals.plos.org/plosone/s/data-availability*](http://journals.plos.org/plosone/s/data-availability)*.*

*Upon re-submitting your revised manuscript, please upload your study’s minimal underlying data set as either Supporting Information files or to a stable, public repository and include the relevant URLs, DOIs, or accession numbers within your revised cover letter. For a list of acceptable repositories, please see* [*http://journals.plos.org/plosone/s/data-availability#loc-recommended-repositories*](http://journals.plos.org/plosone/s/data-availability#loc-recommended-repositories)*. Any potentially identifying patient information must be fully anonymized.*

*Important: If there are ethical or legal restrictions to sharing your data publicly, please explain these restrictions in detail. Please see our guidelines for more information on what we consider unacceptable restrictions to publicly sharing data:* [*http://journals.plos.org/plosone/s/data-availability#loc-unacceptable-data-access-restrictions*](http://journals.plos.org/plosone/s/data-availability#loc-unacceptable-data-access-restrictions)*. Note that it is not acceptable for the authors to be the sole named individuals responsible for ensuring data access.*

*We will update your Data Availability statement to reflect the information you provide in your cover letter.*

We have updated our data sharing statement to state:

Data cannot be shared publicly because of legislative restrictions on the use of registry data from the Australian Orthopaedic Association National Joint Replacement Registry (AOANJRR). Data are available from the AOANJRR for those who meet the criteria for access. External access to and use of de-identified AOANJRR data and CRISTAL data is permitted but must be in accordance with AOANJRR policies (Ref No POL.S3.3, S3.4, S3.5) available on the registry website: <https://aoanjrr.sahmri.com/> policies. Requests for data use can be made by contacting the AOANJRR: Telephone: +618 8128 4284 Email: [adhocs@aoanjrr.org.au](mailto:adhocs@aoanjrr.org.au).

This has now been included in the revision submission.

**REVIEWER 1 COMMENTS:**

This is an interesting study to compare aspirin to enoxaparin for symptomatic VTE prophylaxis within 90 days of any type of hip or knee arthroplasty performed for any diagnosis, in patients enrolled in the CRISTAL trial. I have a few comments.

1. *Is this analysis prespecified or post hoc? If prespecified, why did not the authors provide intonation in original JAMA paper? If this is a post hoc analysis, the authors need to state it. Furthermore, current format of manuscript looks like a first report of the CRRISTAL trial.*

The analysis was prespecified as per the Statistical Analysis Plan that was published prior to the publication of the original JAMA Paper.

This has been clarified in the **“Introduction”** section on page 8:

This report expands the scope of the primary study by reporting on a prespecified analysis[6] and including all patients enrolled into CRISTAL undergoing any hip or knee arthroplasty procedure (partial, total, primary or revision) for any diagnosis.

It has also been updated in the **“Trial participants”** section under the **“Materials and methods”** on page 10:

The primary paper was restricted to patients undergoing primary total hip or knee arthroplasty for osteoarthritis. This paper reports a prespecified analysis [6] and includes all enrolled patients undergoing any arthroplasty procedure performed for any indication.

2. *When looking into Table 1 and Figure 1, the numbers of patients are different from the original JAMA paper. The authors should explain how these numbers come.*

We thank the reviewer for this comment.

The number of participating hospitals (31) and the total number of patients recruited from each arm prior to patient exclusions (7238 to aspirin and 5146 to enoxaparin) are the same as those published in the primary paper published in JAMA (August 2022).

However, the total number of patients included in the final analysis for this paper (after patient exclusions) in the aspirin (6901) and enoxaparin (4827) groups versus the number included in the JAMA paper (5416 and 4827 respectively) is greater, as the inclusion criteria were broader for this paper. This paper included all patients enrolled in the CRISTAL trial (undergoing any arthroplasty procedure performed for any indication), whereas the primary paper published in JAMA was restricted only to patients undergoing primary total hip or knee arthroplasty for osteoarthritis, eligible to receive the study drug. This has been clarified in the **“Trial participants”** section under the **“Materials and methods”** on page 10:

The primary paper was restricted to patients undergoing primary total hip or knee arthroplasty for osteoarthritis. This paper reports a prespecified analysis [6] and includes all enrolled patients undergoing any arthroplasty procedure performed for any indication.

**REVIEWER 2 COMMENTS**

*First of all, the Reviewer would like to congratulate the authors for the current interesting study, which could give clinicians useful information for the issue. The primary publication was restricted to patients undergoing primary total hip or knee arthroplasty for a diagnosis of osteoarthritis, and provided a huge impact. This report included all enrolled patients undergoing hip or knee arthroplasty procedures for any indication. Although not as comprehensive as the main paper, this report also provides sufficient important information. The reviewer has no more comments for this paper.*

We thank the reviewer for this comment.

**REVIEWER 3 COMMENTS**

This study presents a second analysis of the CRISTAL trial, examining the effectiveness of aspirin versus enoxaparin in preventing symptomatic venous thromboembolism (VTE) following hip or knee arthroplasty. The primary analysis of the CRISTAL trial, published in [Ref 3], focused on patients undergoing total hip or knee arthroplasty. In contrast to the primary CRISTAL publication, this study includes all types of hip or knee arthroplasty, aiming to improve the generalizability of the conclusions. Conducted across 31 Australian hospitals, the trial was a cluster-randomized crossover, registry-nested non-inferiority study. Patients received either 100mg of aspirin daily or 40mg of enoxaparin daily, with the treatment duration varying depending on the type of arthroplasty. The conclusion from this secondary analysis is that, for patients undergoing any type of hip or knee arthroplasty, enoxaparin is more effective than aspirin in reducing the risk of symptomatic VTE and joint-related reoperations within 90 days. The sample size and power of this study are well-justified. The statistical analysis is appropriate, and the conclusions are supported by the data.

1. *One minor concern is that this study examined five different secondary outcomes and found a significant difference (p=0.03) in one secondary outcome. However, this may be due to multiple comparisons, and the result is likely to be insignificant after adjusting for multiple comparisons.*

We thank the reviewer for this comment. Adjustment for multiple comparisons was not included in the *a priori* statistical analysis plan. We have now addressed this as a limitation in the **“Discussion”** section on page 21:

We also recognise that although the secondary outcome of joint-related reoperation included all relevant procedures (MUA, wound complications, fracture, deep prosthetic infection, prolonged drainage), an analysis by type of reoperation was not performed. Further, this result should be interpreted with caution since five secondary outcomes were analysed without a multiplicity adjustment and the result may be due to chance.

We thank you for your email and the reviewers for their detailed comments and we hope our responses and manuscript changes have clarified any outstanding issues. We look forward to your response.

Yours sincerely,

Dr Verinder Sidhu (corresponding author) on behalf of all authors

Postal Address: Ingham Institute, 1 Campbell Street, Liverpool, New South Wales, Australia, 2170

Email: [verinder.s.sidhu@gmail.com](mailto:verinder.s.sidhu@gmail.com)

Phone: +61 422 167 435
